# Between the Library and Lectures: How Can Nature Be Integrated Into University Infrastructure to Improve Students’ Mental Health

**DOI:** 10.3389/fpsyg.2022.865422

**Published:** 2022-06-17

**Authors:** Francesca Boyd

**Affiliations:** Department of Landscape Architecture, University of Sheffield, Sheffield, United Kingdom

**Keywords:** nature-based interventions, urban nature, student wellbeing, university, place attachment

## Abstract

The university campus provides the backdrop to a student’s education and social journey. For many students, the transition from secondary school through to graduation can be one of upheaval, geographical, financial and social change. Evidence suggests increasing levels of mental health difficulties among UK university students. The university campus is a possible resource to mitigate wellbeing issues through facilitating the salutogenic effects of engagement with nature. This mixed method research examines the opportunity to integrate nature through interventions for University of Sheffield undergraduate students. It uses a green prescription style activity and a specially designed mobile phone app. Through focus groups the participants’ experience reveals the necessity for a whole university approach that considers intervention and campus design simultaneously. This study’s findings qualify research into young adult’s experience of urban green spaces and their tangible connection to plants such as trees. Policy and practice implications include the requirement for a coherent approach to understanding the place-attachment aspects to nature in the university environment. Further afield, there is a need for collaborative wellbeing interventions and urban green space development within the UK context.

## Introduction

The university campus provides the backdrop to many young adult’s experience of higher education, living away from their parents and discovering more independence. It is a time of transition acted out in front of the landscapes and spaces of libraries, student unions, academics offices and green spaces between bus stops, deadlines and lecture theatres. University campus design varies depending on location, history and estate, ranging from historic Capability Brown landscapes (Bath Spa University, 2016) through to multiple locations in dense urban cities (King’s College London, 2021). The experience of and opportunities to engage with green space will greatly differ between campus type. The University Mental Health Charter identifies the physical environment as pivotal in creating a supportive environment for the promotion of mental health (Hughes and Spanner, 2019). As the determinates of health reach beyond the influence of medical interventions, landscapes form part of the consideration of social, environmental and cultural aspects of health (Barton and Grant, 2006). There is a need to take a holistic approach to the transitional experience of university and understand the environmental and educational impact on student wellbeing (Cage et al., 2021).

### University Student Experience

Students who suffer from mental health issues are more likely to drop out of university, underperform academically and less likely to secure higher level employment (Office for Students, 2019). Emerging research into the role of nature and wellbeing has highlighted the opportunity for the university campus to provide a salutogenic influence, as natural environments provide restoration to university students and are a contributing factor to student retention (McFarland et al., 2008; Windhorst and Williams, 2015). Participants favoured locations which allowed for a separation from everyday pressures. Specifically, and in a change to the usual narrative on green space, this study found the lack of social interaction in the space was important. The natural environments allowed participants to be away from the social expectations and perceived social judgement within university life (Windhorst and Williams, 2015).

Two studies of the perceived greenness of university campus and student wellbeing in the USA and the UK found that greenness was significantly associated with student quality of life and the restorativeness of the campus environment (Speake et al., 2013; Hipp et al., 2016). In Hipp et al. (2016) USA study, the pathway between quality of life and greenness was mediated by the perceived restorativeness of the campus. They conclude that green spaces on campus provide restoration during the stressful life transitions which occur while at university (Hipp et al., 2016). This finding is furthered by Holt et al. (2019) research that found those undergraduates who regularly engaged with the natural environment through regular physical activity reported higher quality of life, positive emotions and lower perceived stress. Jones (2013) introduced the root model of a biophilic university, the idea being to provide spaces allowing for the restoration of affinity with nature. Thus, campus environments that provide access to nature offer economic, social and health benefits for those studying and working on campus (Jones, 2013; Astell-Burt and Feng, 2019; Campisi et al., 2022). Overall, students’ intrinsic and extrinsic experience of academia and the university campus environment are associated with academic accomplishment (Liprini, 2014; Hipp et al., 2016; Hughes and Spanner, 2019). The open space which surrounds the university buildings provides alternative spaces to work, socialise and relax (Liprini, 2014).

### Facilitating Moments in Nature

In a progressively urbanised world, there is a particularly important role for nearby nature (Kaplan, 1993). Momentary, incidental or indirect contact with nature such as a view from a window or noticing a street tree may provide micro-opportunities for restoration (Maller et al., 2009). While the direct mechanisms behind the effects of nature on health and wellbeing still require further exploration, there is consensus within the evidence base that green spaces in urban environments provide multiple health benefits (Frumkin et al., 2004; de Vries et al., 2013; Hartig et al., 2014; Panno et al., 2017). Urban green spaces contributing to these benefits include large and small public parks, pocket green spaces, trees along a street or parklets which provide a place to relax created through plants and seating which are located in a place usually allocated for car parking (Astell-Burt and Feng, 2019; Campisi et al., 2022).

As with direct engagement, the benefits gained by an individual’s indirect engagement with nature are reliant on the individual’s preference, perceptions of and experiences within natural environments (Hartig et al., 2014). Evidence demonstrates differences in responses towards natural environments experienced by different demographic groups. Cultural and socio-economic background, gender and age affect an individual’s response to the natural environment (Dallimer et al., 2014; Boyd et al., 2018; Hughes et al., 2019). Nature connectedness is an individual’s subjective sense of their relationship with nature (Pritchard et al., 2019). Additionally, studies support the effect appreciation of the beauty of nature as a factor in increasing nature connectedness (Zhang et al., 2014; Richardson and Sheffield, 2017). Specifically for university students, this connection has been found to influence sustainability behaviours and wellbeing (Redondo et al., 2021).

While the implementation of urban green infrastructure is important, it is not truly effective without complimentary social initiatives. A recent meta-analysis evidenced the social, economic and health outcomes of urban green infrastructure, ranging from green walls through to initiatives promoting green trails (Hunter et al., 2019). This analysis found strong evidence to support interventions implemented alongside promotion of programmes in parks and green trails. The combination of improved urban design alongside social intervention to promote physical activity and community initiatives, created a more effective response from the population, as evidenced in the increased use of the areas and physical activity (Hunter et al., 2019). This exemplifies the importance of collaborative working by all agencies involved in urban planning and health initiatives of this kind. Green urban infrastructure requires a holistic partnership across multiple agencies to be sustainable and efficacious.

### Knowledge Gap

The prevalence of mental health conditions among university students provides motivation to facilitate better wellbeing through the benefits afforded by the natural environment. Previous research methods have involved questionnaires, simulated environments or interviews with university students to gain a theoretical understanding campus green space impact. The importance of university green space for student wellbeing and success has been identified. However, there is limited knowledge encapsulating the variety of experience when in the green space, and a lack of measured outcome effects from visiting these spaces (Speake et al., 2013). The majority of studies has focused on perception and preference for green space characteristics rather than monitoring the effect of visiting these spaces through measurable outcomes or discussion on preferred design features.

It is known from research on place-making and place-belonging that the practices which occur as part of the identity discourse differ between location and community (Benson and Jackson, 2013). The identity of a place is created, in part, through the intersection between behaviours and the unspoken narrative which exists within a community (Scannell and Gifford, 2010). This epistemology is applicable to our perception of green space; for example, visiting the park during lunchtime might have negative influences on a person’s professional image, scuff their suit, or affect a colleague’s perception of their work ethic (Hitchings, 2013). Previous research focused on workplace green space offers similar insights into how places operate under similar built physical infrastructure and social pressures to university campus. This nuanced difference in physical infrastructure and the behaviour conducted there provides evidence of a knowledge gap in relation to university students’ experience.

## Materials and Methods

The research’s methodological approach is founded in place-making and place-belonging which examines the participants interactions and experience of the university campus and the designed interventions. It also draws on Lumber et al.’s (2017) experiment exploring responses to nature-based activity as a wellbeing intervention in small groups with pre- and post-metrics. This research uses a mixed method approach, with the statistical analysis and focus group discussion forming an overview to the participants experience. In particular, focuses on the landscape design elements of the qualitative research. Drawing on a grounded theory approach to novel data emergence, a secondary research question was development in response to the direction of discourse being presented by the participants.

### Research Question

1. How did the participants experience the interventions?

1a. What are university students design preferences for campus green spaces?

Aspects of the author’s PhD focused on other research questions, with the research question above (1) being the initial focus for this study. Research question (a) emerged over the course of the research as participants discussed their experience of urban green space in Sheffield and at home, during the group walks and focus groups.

### Sampling

Recruitment for this study aimed to be representative of the student population and avoid recruiting those already engaged with nature, perpetuating the knowledge gap regarding those with limited nature connection. To reduce this bias, recruitment was advertised as an ‘urban green’ research project rather than ‘urban nature’ or ‘nature engagement’. Recruitment occurred through the university research participants email list, flyers distributed across the campus and through direct contact with University of Sheffield societies. There was an initial valid expression of interest from over 200 students. An exclusion criteria and collection of basic demographic information allowed the research to focus on those most likely to be undergraduates (age 18–24 year old).

The second wave of recruitment and intervention for group 2 and 3 (involving the walk) was needed to mitigate for the high dropout rate (~60%) which occurred after the initial wave of recruitment. The second wave occurred the following week to reduce change in environmental conditions and not clash with the Easter holidays.

### Intervention Design

**Figure 1:**
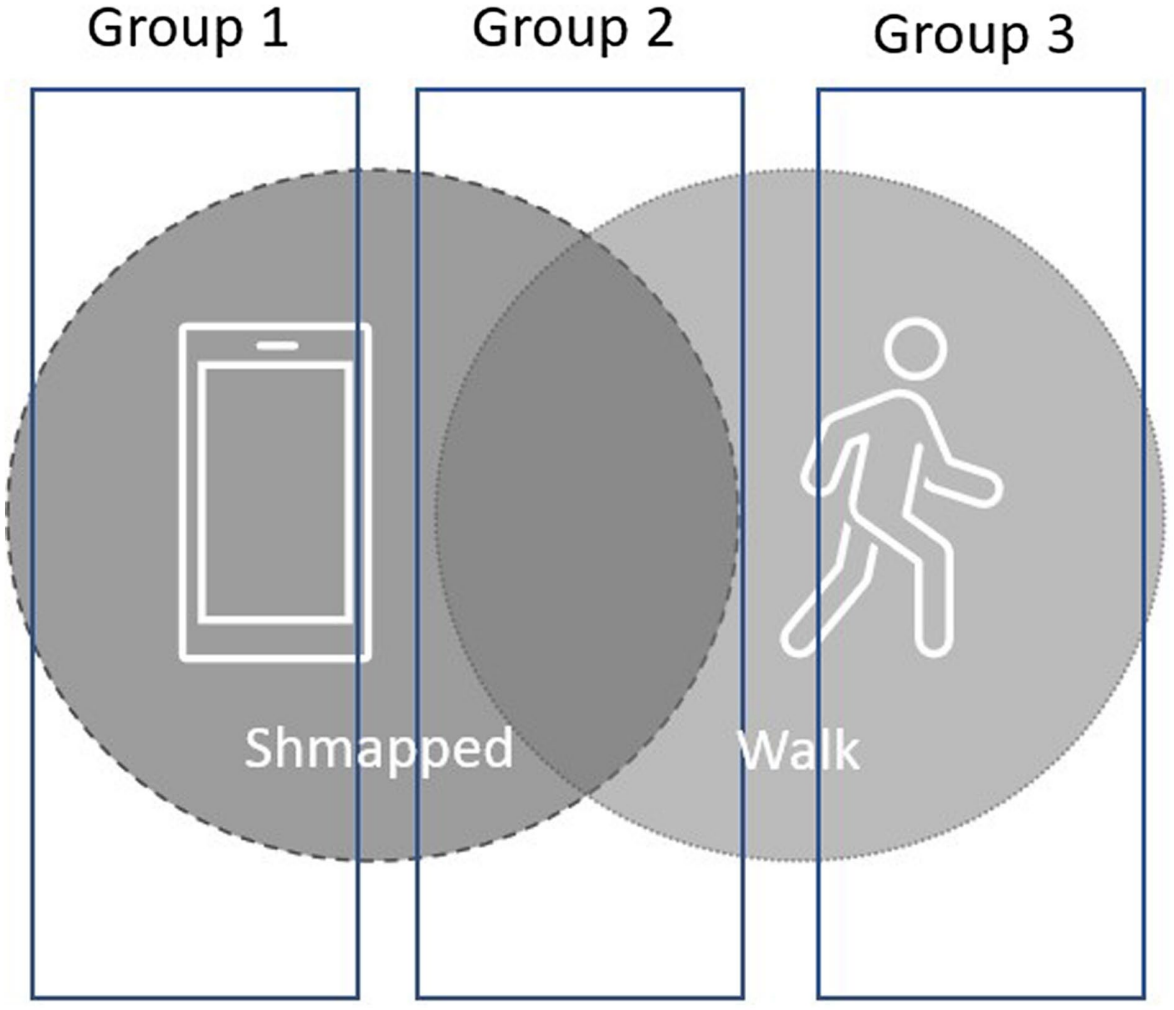
Overview of method.

This study contained two interventions (detailed further below): a mobile phone app called ‘Shmapped’ and a walk intervention. These formed three conditions: (1) Shmapped App only group, (2) Shmapped App and Walk group and (3) Walk only group (see Figure 1).

It should be noted that during this time there was a strike by university staff which stopped teaching on campus and unprecedented heavy snow. Both are likely to have affected the study but also reflect the varying nature of university life for students.

#### Mobile Phone App

**Figure 2:**
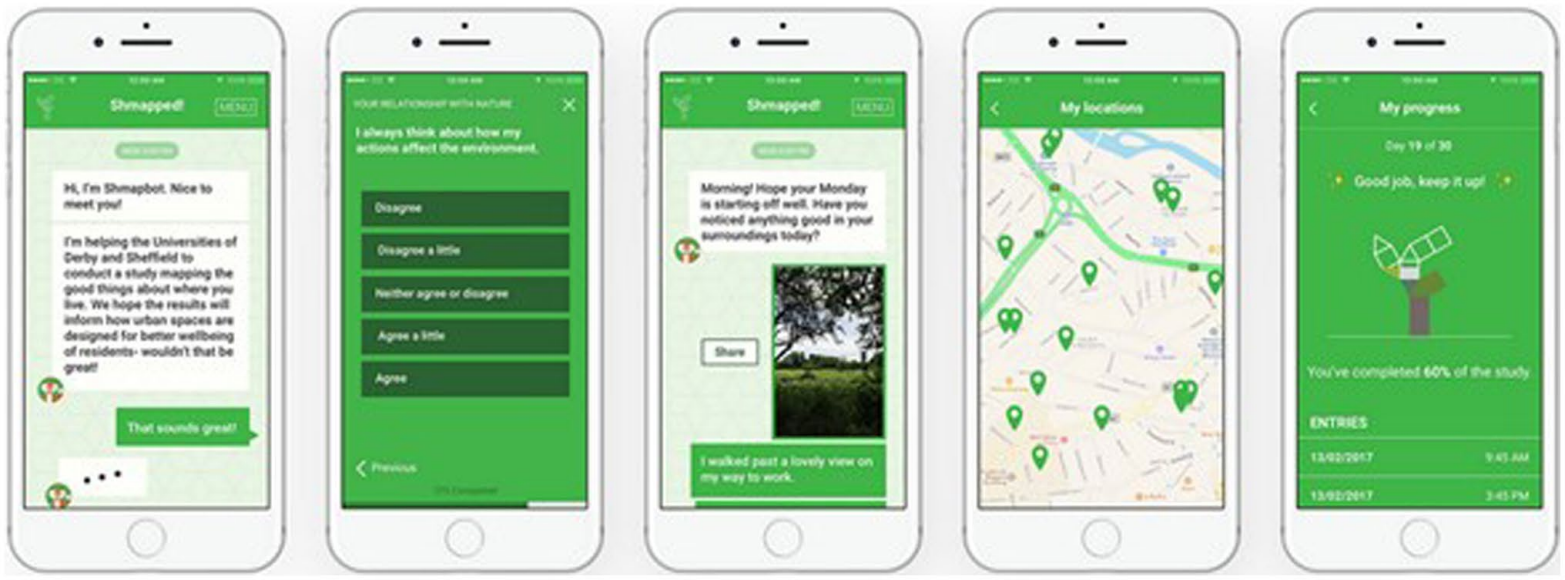
Example of Shmapped app interface.

The app ‘Shmapped’ (Sheffield—Mapped) was developed and inspired by research conduct by the IWUN project team (Richardson and Sheffield, 2017). Shmapped was designed in collaboration with the app development company ‘Furthermore’ and included a user development test group (McEwan et al., 2020). The mobile phone app functioned as an intervention and a research tool for data collection. The research tool part ran as a background function to the daily intervention notifications. The front house function of the app displayed as a chatbot fox which asked the participants questions in relation to noticing nature daily (Figure 2). The app collected the before, after and follow-up metrics. Shmapped was only available on Android and Apple phones, excluding participants without smartphones or using a different operating system.

Initially designed as a 30 day intervention, uptake and adherence was poor with only 55 participants completing the full 30 days. Therefore, the app was redesigned to be 7-day intervention with the measures at baseline, post 7 day and 30 day follow-up. The redesigned intervention ran throughout Winter 2017/Spring 2018 (McEwan et al., 2020).

#### Walk

Designed to replicate green prescription walk activities and encouraged participants to meet the regular 20–30 min in nature threshold (Tyrväinen et al., 2014; Shanahan et al., 2016; Active Fife, 2019; Hunter et al., 2019). The walk intervention composed of a group walk at the beginning of the week followed by a solo walk at the weekend. The walks aimed to provide an appropriate break in the participant’s day and enough time in a green environment for them to receive restorative benefits (Hartig, 2006).

##### Group Walk

The walk was located in an accessible local park. Four different time options were offered per group, this resulted in a total of seven walks being undertaken with the first wave of participants. The group walk was designed for a small group of up to five participants. This was to support social engagement while being sensitive to the other users in the park. In practice group, size was unpredictable with timetable changes and cancellation effecting attendance. This resulted in group size ranging between one and seven.

**Figure 3:**
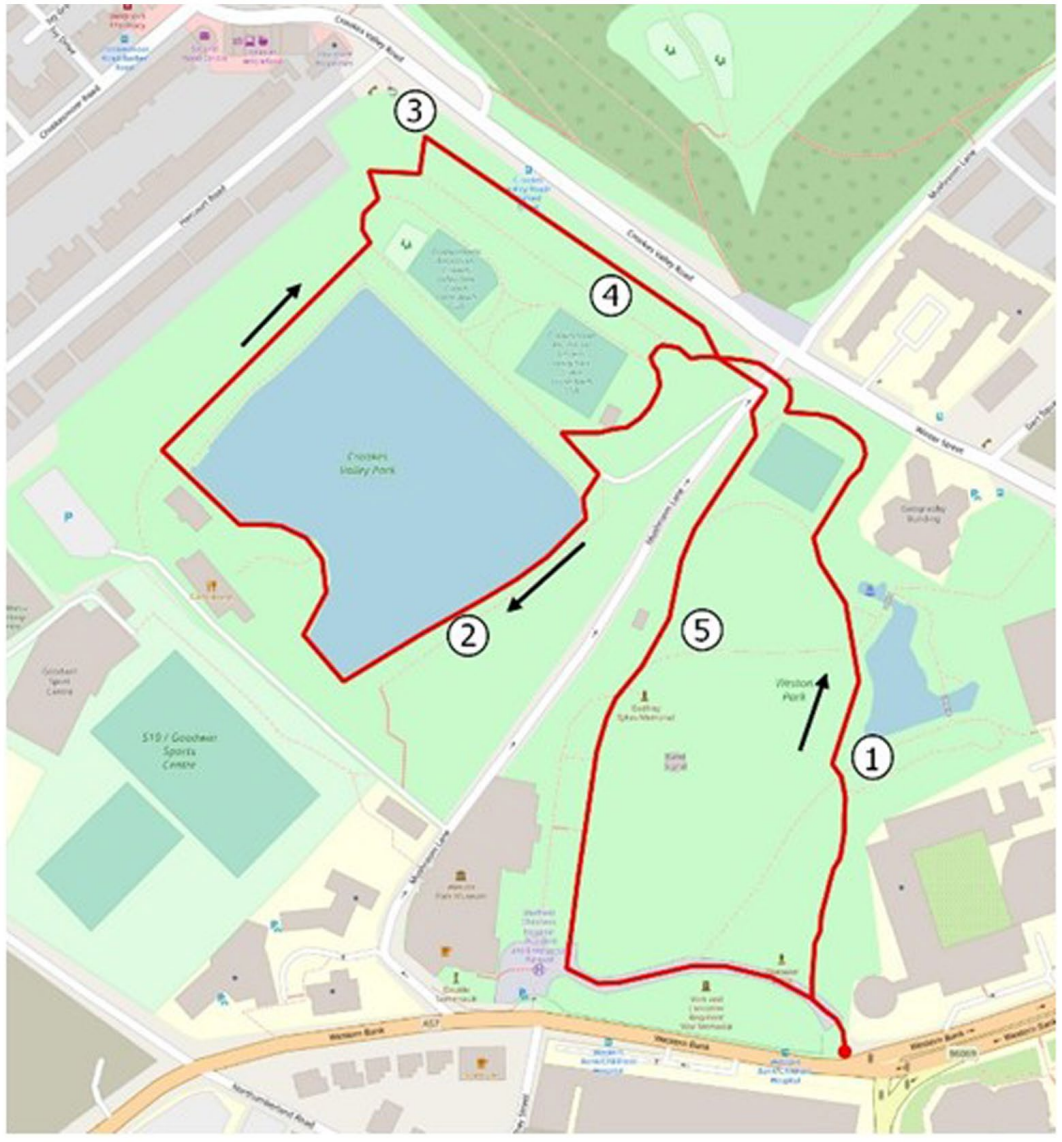
Map of walk route in Sheffield, United Kingdom.

The walk travelled through two local public parks (Figure 3). Weston Park is 5 hectares with the boundaries defined on three sides by roads. A municipal park opened to the public in 1875 it retains much of its original planting scheme. The wide expanse of grass includes tennis courts, monuments and an irregular shaped pond (Historic England Archive, 2004). Crookes Valley Park was created around the existing reservoir in the early 20th century. The central feature is the Old Great Dam built as a water reservoir in 1785 (Friends of Crookesmoor Parks, 2020). The park developed over the past century to include a pub, bowling green and a children’s play area. It is just under 5 hectares and contains an area of naturalistic woodland with occasional rose flowerbeds.

As the facilitator, the first author had scripted verbal prompts to direct the participants attention to different elements of the walk. Identified in Lumber et al (2017) work and structured around the nine values of the biophilic hypothesis, the pathways include as: contact, emotion, meaning, compassion and engagement with nature beauty. These prompts were designed to encourage connection with the natural environment. Language when discussing nature was kept non-technical to support accessibility.

##### Individual Walk

The second walk was under the participant’s own initiative and aimed to encourage the participants to walk for over 20 min and to use it as an opportunity to explore a new place. Participants were sent an email reminder on the Friday.

### Data Management and Analysis

The project has been ethically reviewed by the Department of Landscape in accordance with procedure laid down by the University of Sheffield’s Research Ethics Committee, which monitors the application and delivery of the University’s Ethics Review Procedure across the University, reference number: 016529 and 014504.

**Figure 4:**
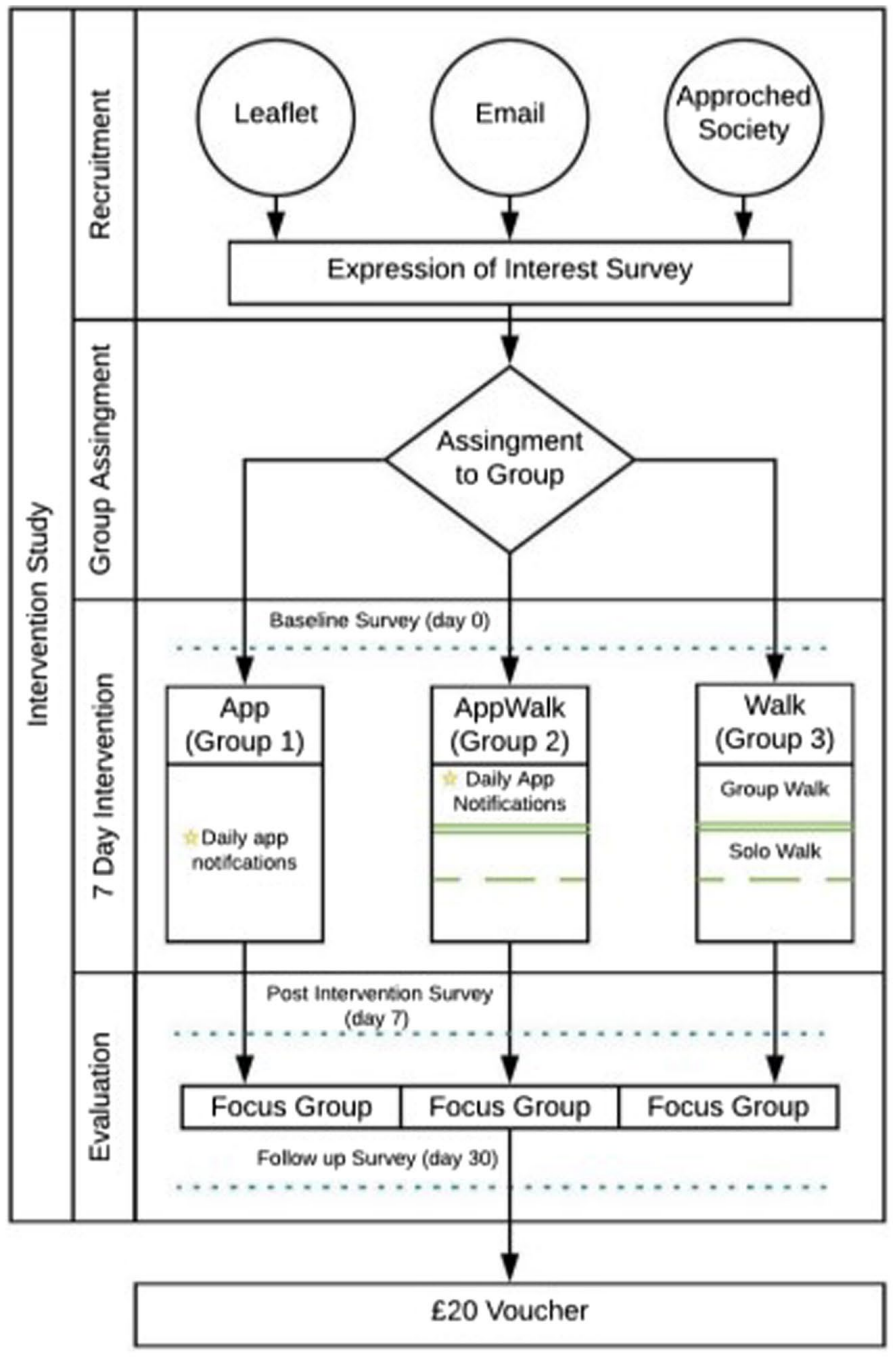
Intervention design.

Data were collected at day zero (pre intervention), day seven (post intervention) and day 30 (follow-up; see Figure 4). The question format was designated by the Shmapped design and replicated for the non-app users *via* an online survey. The following metrics were collected as: Recovering Quality of Life (ReQoL), Nature Relatedness (NR-6) and Inclusion of Nature in Self (INS; full description is available in the Supplementary Material). For all the measurements excluding INS, the responses are on Likert scale. The analysis of these results from these can be found in an article published elsewhere (author, in review).

It was important to evaluate the experience of the intervention from the students’ perspective. A challenge in green prescriptions and student support service is attendance and adherence. The experience of the participating students was collected through focus groups.

### Focus Groups

Across nine 1-h focus groups, 26 participants discussed the intervention and their experience of engaging with the natural environment which at university. Participants’ background varied in ethnicities, ages, course studied, year of study and gender. Recruitment to the study initially allowed for the groups to be representative of the undergraduate student population at the University of Sheffield. However, due to drop out, this was not maintained. The set questions were based on the app user’s experience and separately on the walk experience. It was important that the experiences of the intervention were allowed to be articulated by the participants and that these experiences were considered within the data analysis. Therefore, a grounded theory approach was taken. As the data accumulated the researcher continually assessed and then adapted some of the questions to identify discrepancies and commonalities in the data. This resulted in the green space questions evolving to included conversation on preferred spaces for socialising and taking a break.

#### App User Questions

Group one and two included questions on the usability, design, visual appeal and different features of the app. To gauge the apps application outside of the research study with university students, participants were asked if they would recommend it to a friend. This section of questions included opportunities to discuss improvements and limitations.

#### Walk-Related Questions

For groups two and three, the use of drawing the group walk and park got participants to recall the walk and generate discussion. Once they had created their group drawn map of the walk, participants were asked to mark any area they particularly liked or disliked, sensory elements they may have remembered and if the areas were familiar. Participants were asked to describe the individual walk they went on including, if it was part of their usual routine or new to them. From this topic, participants discussed different areas within Sheffield that they enjoyed or avoided walking through (Boyd, 2022).

#### Green Space Questions

The questions were designed to be flexible and allowed the discussion about experiences of nature on campus. As is important with focus groups, while the facilitator offered the topic, the conversation was allowed to develop between participants. Recent infrastructure work on campus and rumours of converting a large carpark into a green space, provided an opportunity to discuss participants’ preferences. The carpark is a large space located between the student union, a library and three other departmental buildings. The recent urban infrastructure was outside the Diamond building (opened 2015) which is central space for studying and teaching across disciplines.

### Transcription and Coding Process

**Figure 5:**
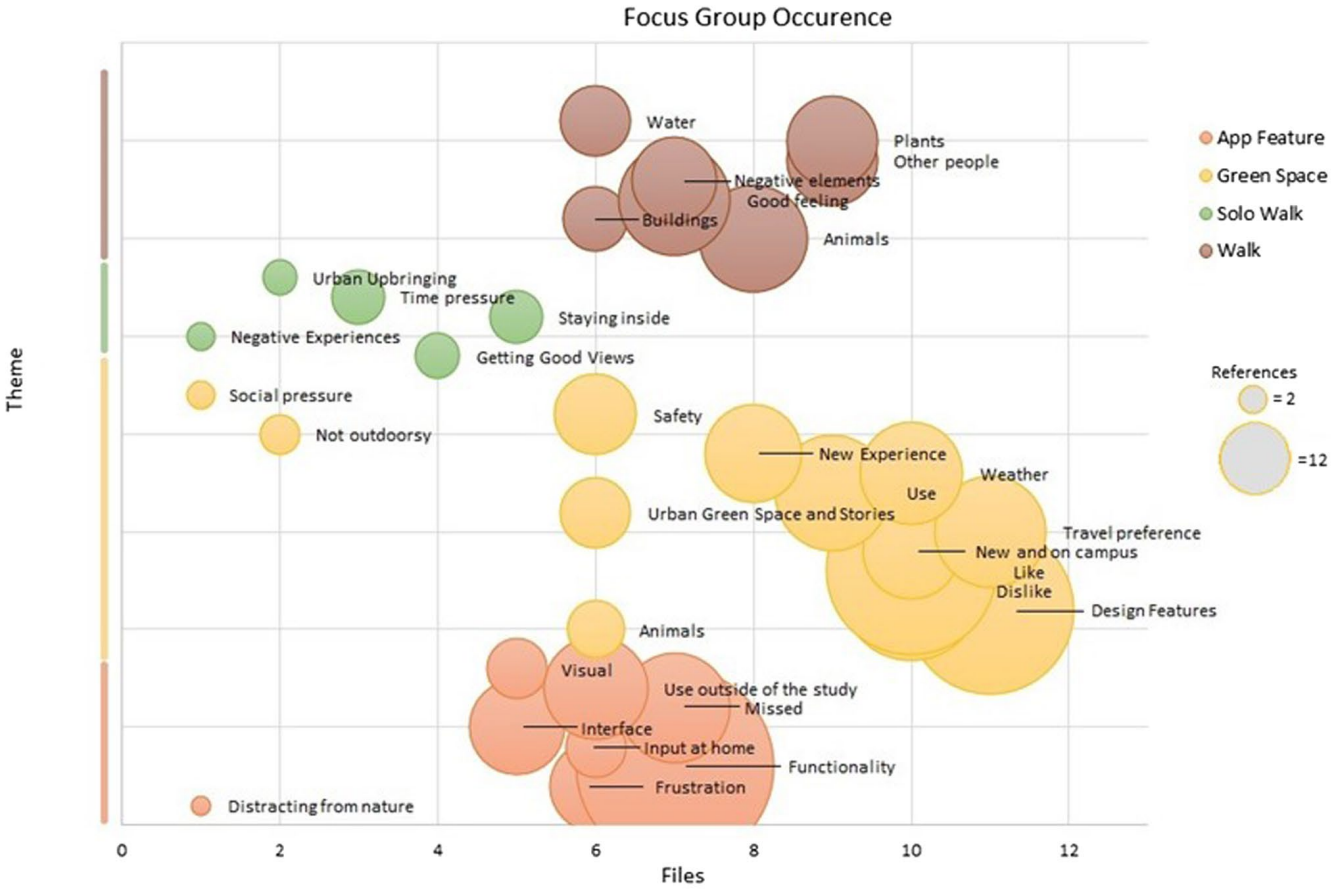
Focus group theme codes.

The focus groups were digitally recorded, anonymised and transcribed. Focus group transcriptions were coded through NVivo (version 12) to identify key priorities for participants from a nature-based intervention and developing opportunities to engage with nature on campus. These codes are kept as close to the original context as possible to allow the themes to organically emerge before being related to one another and pre-existing theories (Charmaz, 2006; Sbaraini et al., 2011). Transcriptions were coded into broad categories and then additional shared themes were created as dictated by the data as it accumulated. As with best practice in grounded theory, the coding went through two stages; an initial stage in which themes emerge inductively and a second stage of focused coding which pursues a central set of codes. This was achieved by an initial coding of all themes within the dataset before being refined into final categories which are central to the entire study and relate to one another (see Figure 5).

Grounded theory emphasises the relationship between coding and emergent themes. Charmaz (2006) summarises this as ‘coding is the pivotal link between collecting data and developing an emergent theory to explain these data. Through coding, you define what is happening in the data and begin to grapple with what it means’ (p.45). It was important that the full experience of the intervention was considered, as without a detailed understanding of the range of experiences the challenge of uptake and adherence would continue. The key themes discussed in this paper emerged from the detailed exploration of the data without pre-defined boundaries to coding. As seen in the discussion, these codes were compiled to form hypothesis on the role of intervention and furthermore infrastructure. Ten percent of the transcriptions have been checked by a second (blind) researcher to monitor for bias in attributing codes to themes. Additionally, the two-stage approach to the coding process supported a comprehensive understanding of the content within the focus groups.

## Results

The focus groups brought forward specific themes in relation to urban green space design and their experience on campus (see Table 1). Participants expressed concern over the safety of some urban green spaces, including ideas that areas were dangerous because of police cars sightings. The results relating to the unpleasant and unfamiliar aspects of urban green spaces have been published elsewhere (Boyd, 2022). There was extensive discussion on the social constructs related to different urban green spaces in and around campus. Including the idea that some areas ‘belong’ to a department. The most frequent and broad code was the elements participants liked such as flowers in bloom and trees providing shelter. Of equal occurrences were the aspects of design features, these were predominately new design features that participants would like to see on and around campus, such as ponds, picnic benches and winding paths. In third comes, the participants disliked such as litter, geometric design and manicured lawns. The full list of codes and quotes is available and accessible in the author’s thesis (Boyd, 2020).

**Table 1:** Occurrences of codes in relation to green spaces with illustrative example quotes.

Green Space	Code occurrence	Example quotes
Animals	**8**	*I felt warm while watching the ducks, pigeons, pets, colourful flowers*
Design Features	**67**	*some rocks and then like there’s a little pond fountain thing, just a little one, doesn’t have to be a lake, just one to the side, like a tear fountain*
Dislike	**47**	*because flowers on there, it feels unnatural, like someone’s planted them and yeah it’s pretty but it doesn’t feel natural*
Like	**68**	*I think I also appreciate, trees, just trees a lot of, a bunch of trees like in this place*
New and on campus	**22**	*I felt very relaxed during the walk, probably again because of the company, although the escape from there busyness of the city certainly helped*
Travel preference	**30**	*I like would not walk down there either, like heard that […] mushroom lane is quite a dodgy at night*
Urban Green Space and Stories	**12**	*You know I think you're asking for trouble if you're walking through a public green space if it’s dark and late at night*
Use	**32**	*sometimes you want to be able to sit on a bench instead, maybe even like picnic tables or something because sometimes benches can be a little bit anti-social if there’s quite a few of you*
Weather	**25**	*Some shelter if it’s a sunny day or rain if you want to sit there in the rain.*
New Experience	**23**	*I’ve walked past Crookes Valley Park but I’ve never been in it [before the research]*
Not outdoorsy	**4**	*I’m not very in tune with nature I’m a much more urban person*
Safety	**16**	*and even like Western Park which is like right on the main road you see police cars there and stuff all the time so its like I don’t really want to go in there.*
Social pressure	**2**	*bench where you don’t sit in a row […] they are very unsociable*

The previously mentioned codes can be understood in a more contextual factor as the grounding for five elements of university students engagement with urban nature. These five elements are presented here alongside participant’s quotes and discussed later in relation to the literature and place attachment.

### Experience and Design of University Spaces

There was an incoherence between participants’ experience of indoor and outdoor space design on campus. Recently built urban green spaces were found to be too busy as the benches and planters were features of a cycle and walking route.

*‘What do you guys think about the path, the bench and stuff on the other side? With the trees near it because I don’t know how like, I wouldn’t really want to sit there because everyone is always like running past you like’.*

Participants identified a lack of outdoor spaces that felt green (contained nature) or felt that certain green spaces were for department specific students.

*M: I don’t know if I’ve been to any that actually I’d call green places*

*FN: Nor me actually.*

*‘I really like Firth court it is kind of my building but it’s just nice as you go through it’s got a massive staircase, nice entrance then in the middle there is a courtyard it’s just pretty, calm’.*

When thinking about the possibility of new urban green spaces, participants were interested in places which facilitated their priorities around studying and socialising. Often the type of spaces found in the library or student union but recreated with trees, benches and shrubbery.

*‘Mhmm well I guess make it more of a meeting space for people. Which would also need to incorporate some greenery and spaces and structures that can be used for a variety of things, as part of the built landscape but you can use built structures or whatever to sit or stand or lean on or anything like that and also areas where you can meet, like under this tree or this post’.*

### Socially Constructed Elements of Green Space

There was a strong desire for sociable spaces. A place to eat lunch, meet friends and relax away from work. This was often expressed through the desire for seating, particularly benches which were not in a straight line. As expressed by these participants:

*KB: I think like benches but erm bench where you don’t sit in a row. as they are very unsociable*

*F: facing each other?*

*KB: yeah that why I think I like picnics benches are attractive and yeah like nice flower plants.*

The urban green spaces on campus were heavily influenced by participants perception of danger or cultural expectations. For example, an idling police car suggesting crime or a decommissioned cemetery retaining cultural social significance.

*‘and even like Western Park which is like right on the main road you see police cars there and stuff all the time so its like I don’t really want to go in there’.*

*‘for me it’s weird seeing people who just walk on the grass around graves and stuff because we have like erm, a how to say, we have respect so we have to avoid stepping on the ground near graves’.*

### Shelter From the City

Sheffield is a large busy city, the campus is situated close to some major roads and hospitals. Participants reflected on the noise and busyness this creates by requesting trees as a way to shelter from the sights and sounds of the city.

*‘And you feel sort of, you can escape into there […] As long as its also like, you know surrounded by something. Maybe trees or you know like in Firth Court’.*

*‘I think we could use trees for a shield for the city. So you see as little building as possible if that makes sense? Because if you have, if you walk along and you’re like oh I can see the trees and oh I can see the arts tower at the same time. that’s why I like Graves Park because its so big and there are so many layers to it you get to the middle you can’t hear, you can’t see anything [urban]’.*

### Wildlife and Wild

The desire for wildlife and wildness occurred in two forms, first as a direct requestion for more plants, variety and landscapes, and second as a discontent for green spaces which were perspective as heavily managed. For example, the previously mentioned Victorian designed Western Park.

*M: Lots of trees.*

*FN: Water, water.*

*M: Oh yeah, preferably wildlife as well as water. Lots of benches because I think, I like sitting on the ground but for when it’s a bit damp.*

*‘it’s really like everything is cut clearly and this stuff is really clean for nature. It’s really artificial’.*

*‘It feels like somebody designed western bank [park] with like a geometry set’.*

### Attitudes to Visiting the Natural Environment

While the predominate narrative was a positive response to more nature and there was a minority (15%) of participants who did not consider themselves to have a proactive attitude towards visiting the natural environment. These participants cited the demand of university work or an urban upbringing as a background to their lack of engagement with the natural environment.

*‘To be honest I haven’t left my house much with the strikes’.*

*‘I don’t appreciate the greenery, if you said me to, look how pretty the flowers are, yeah I see that but it doesn’t change my day. It doesn’t matter to me that I’ve seen like colourful flowers and green plants’.*

*P: See I would say I’m not very in tune with nature I’m a much more urban person, so erm, I mean there were obviously definitely trees around and stuff but there wasn’t anything that stuck out to me there wasn’t like a big tree or anything*

*P: no and especially like people who are from here, they do like walking dates*

*F: do they?*

*P: apparently so, but honestly I don’t know what the aim of this is, I’m having to constantly think of things to say and it’s like why are we walking! I need a destination basically; I can’t just be walkin’.*

The role of unseen dimensions and cultural behaviours contributed heavily to the lack of engagement with urban green spaces. The participants talked at length about their love of trees; however, their desire to stick within the status quo and lack of integrated green spaces reduced their opportunity to interact or visit these spaces while on campus.

## Discussion

The methodological grounded theory approach to allow concepts to emerge over the course of the focus groups which created some interesting and unexpected themes. There is literature detailing the social influence on green space especially among young adults, the experience of university students’ and green space, and the socially constructed elements of green space. However, the strong desire for shelter from the city to have ‘wild’ spaces and the acute awareness of the Sheffield street tree issues was not expected. These six aspects are discussed further, before considering these findings implications for policy.

### Place Attachment

The role of the place became very apparent in how the participants experienced the intervention. The bond which occurs between an individual and their meaningful environment is known as place attachment (Scannell and Gifford, 2010). This bond is associated with pro-environmental behaviour in natural environments and positive psychological benefits such as a sense of belonging or relaxation (Halpenny, 2010; Scannell and Gifford, 2017). The individual connection with place is a dynamic and complex relationship, influenced by social interactions, personal identity and the experience of the physical place (Raymond et al., 2010). The place-attachment framework by Scannell and Gifford (2010) defines three dimensions to the person dimension of place attachment; person-process-place. It encompasses the influence socially constructed narratives have on behaviour and emotional response to an environment or location (Scannell and Gifford, 2010). The role of process and person can be evidenced in the experience of young adults and natural environments (Bell et al., 2003; Milligan and Bingley, 2007). In contrast, the natural environment can be a place to escape to, with teenagers reporting the more unkempt spaces providing a place of peace without judgement (Bell et al., 2003). These elements may present themselves differently for a young adult compared to an employee or visitor to a space. Beyond the physical elements, a space is constructed by individual, social and behavioural dimensions, and these unseen dimensions contribute to the way a space is experienced and used (Raymond et al., 2010; Scannell and Gifford, 2017).

#### Experience and Design of University Spaces

In the case of university open spaces, focus group participants reported that the perception of those spaces had a greater influence on the way those spaces were used/not used and the benefits derived from them than the reality of the space themselves (Beckers et al., 2016; Hipp et al., 2016). This aligns with research on study space design for university students, which highlighted that the perceived value of a space was more important than its experienced value (Beckers et al., 2016).

Previous research has suggested that the campus environment should be designed to have open spaces which create an integrated blend of sheltered spaces for study and open spaces for collaboration (Beckers et al., 2016). These spaces should be clearly defined to denote expected behaviour within the space and so reduce the stress that can occur when a space is not coherent (Lau et al., 2014). The desire for collaborative and sheltered spaces was qualified through this study focuses group findings. In alignment with this and others’ research, campus design is emerging as a potential wellbeing component of the university experience (Hipp et al., 2016). Previous research has considered the biophilic campus, campus design to integrate sustainability and promote learning and collaboration (Ibrahim and Fadzil, 2013; Matloob et al., 2014; Abdelaal, 2019). Future research into campus design could take these ideas further by working in collaboration with the users’ perceptions and lived experience of campus green space. Through the focus group discussion, this research found three key dimensions of importance:

#### Socially Constructed Elements of Green Space

There are attributes in the design of urban green spaces which impacted the participants ability to engage with a space (Seaman et al., 2010; Bell et al., 2014). The complexities which surround a green space on campus are enwrapped in the socially constructed narratives and personal preference. As discussed by Bell et al (2014), these personal preferences are susceptible to change as influenced by circumstantial priorities and place practice.

In the focus groups and through the survey feedback, the social narrative surrounding the risk of entering urban green space in the dark was reflected across nationalities, age and gender. These university students had heard stories related to incidents on campus or had personal experience of them. Urban green spaces being considered dangerous at night heightens the argument for providing accessible green spaces that are appealing during the day, as well as reducing students’ fears to use campus space outside daylight hours. Most of the university term occurs in the less climate favourable time of year between September and April. Daylight hours and weather conditions can reduce the opportunities to engage with the natural environment outside of university time. A prime time opportunity is lunchtime, which participants discussed as having limited current potential for visits to urban green space as the spaces on campus where they currently eat their lunch consist of various ‘grey’ concrete steps.

#### Shelter From the City

Urban green spaces can offer respite from the city soundscape and busyness of campus (Windhorst and Williams, 2015). As found in other research, participants valued the opportunity to feel protected from the sounds and sights of the city (Birch et al., 2020). Previous research has found participants reported feeling calm and relaxed by the presents of water and mature trees (White et al., 2014; Windhorst and Williams, 2015). These restorative aspects of green space visits were acknowledged in the focus groups by several participants who had attended the group walk. This was highlighted particularly in the desire for design features that provided sensory reoccupation such as water fountains and large trees. These participants were also likely to choose a seat by the window in the library to look at the park. In contrast, the mobile phone app only users did not comment on how restored they felt after the intervention. If walking through campus provided a restorative experience similar to walking through the park, it could support better mental health. McDonald et al. (2018) argue for integrating green prescriptions and city designs which harness nature into urban development. Therefore, this research suggests that university campus green space design should be in coordination with interventions, such as introducing green trails alongside cycling schemes.

#### Wildlife and Wild

Unexpectedly from the author’s perspective, focus groups participants all talked about animals and wildlife found in the urban green spaces with affection. Some participants wanted to see wildlife beyond just pigeons, and this could represent a desire for more biodiversity within the spaces they visit. In agreement with this finding, evidence does suggest the role of perceived nature to have a strong influence in the restorative effect of the space, with those with higher nature connection more perceptive of flora and fauna diversity (Hipp et al., 2016; Southon et al., 2018). As previously suggested in the literature, the connection created with city wildlife provides a vital relationship (contributing to pro-environmental behaviour) which can affect the global ecosystem (Dunn et al., 2006).

There was also an attention to the management of landscape features in the urban green space. Some focus groups participants were strongly opposed to intense ground management. This was particularly in reference to the Victorian planting style scheme and manicured grass found in Weston Park. Crookes Valley Park’s area of naturalistic woodland was commented on for offering tranquillity and cover from the city. Wild can be in relation to the perception that nature is dominate compared to where a place looks controlled and maintained (*ibid.*). The influence perceived levels of design and management have on individual’s attachment to a place as ‘wild’ can be replicated in this finding to include difference of preference in a place in relation to its perceived level of design, management and wildness (Colley and Craig, 2019). The different forms and how they are perceived may offer an opportunity to develop established ideas of aesthetic preference, for university students in this research there was a strong preference for less managed environments.

#### Attitudes to the Natural Environment

Young people are often attributed with generational decrease in their connection or knowledge of the natural environment; nature deficit is deemed the result of decreased engagement with the natural environment (Louv, 2008; Moss, 2012). Coinciding with the drop (during adolescence) in nature connection young adults are expected to attribute less importance to the natural environment (Bird, 2007; Hughes et al., 2019; Richardson et al., 2019). While the focus group participants in this study discussed prioritising their studying and socialising (and gave these priorities as reasons for dropping out), there was passion and value for the natural environment. This was most apparent when talking about trees.

As with understanding people’s attitudes towards a physical space, specific elements of the natural environment are also exposed to socially constructed narratives. Sheffield and its trees are an unusual case, as during this research there was a conflict between the local community and the council about street tree management (BBC, 2019). During the focus groups participants in this study spoke passionately about the desire for more, and especially large, trees. Previous research into individually valued restorative space on campus found a positive association with mature trees (Windhorst and Williams, 2015). Specific preferences for different types of plants have not been comprehensively considered within literature on campus green spaces, whereas participants in this research discussed their preference for mature trees, flowering plants, shrubs and natural planting schemes. Further to this, this finding challenges the notion that young people do not value the natural environment, but highlights that they express this in a different way, with alternative unaccounted ways to connect with nature (such as house plants; Birch et al., 2020). Natural England (2019) MENE report identified generational differences in attitudes towards intention to make lifestyle changes to protect the environment. On average 16% of those asked intended to make changes, with young people (16–24 year old) 10% more likely than older people (over 65 years old; Natural England, 2019). This study’s findings qualify research from Birch et al. (2020) in young people’s experience of urban green spaces and the tangible connection to plants such as trees. There is further opportunity for this relationship to be explored within campus and urban green space design.

### UK Policy

Within the context of UK policy, DEFRA’s 25 year environmental plan included the natural environment as a resource for population level health (Defra, 2018). While there is no single government department or body tasked with ensuring the potential benefits between the natural environment and improved population health, many third sectors organisations have begun to acknowledge this within their practice and policy, for example, Mind and the Wildlife Trust (Lovell et al., 2018). The evidence in this research further supports the vital role urban green spaces play in facilitating positive mental health, especially when accessible and of high quality.

### University Policy

While universities compete to be at the top of leader boards for academic attainment, world class research and cutting-edge facilities, it may be time to contemplate the role of the natural environment in supporting wellbeing in the university student experience. Considering the impact of mental health on grade attainment, retention and social cohesion, university campus landscapes could become the next league table. The introduction of a Charter Award Scheme in association with the University Mental Health Charter means that this aspect of the university sector will soon be under closer scrutiny, with an expected assessment and therefore possible comparison as part of the award (Hughes and Spanner, 2019). Universities need to develop proactive, coordinated and strategic approaches to delivering accessible and inclusive wellbeing support that are responsive to the needs of the student population (Priestley et al., 2022). In agreement with other research and as part of the ‘live’ dimension of the Charter, this study has found that university green spaces can be part of that strategy as a wellbeing resource for students and staff (Hipp et al., 2016; Hughes and Spanner, 2019). The University Health and Wellbeing service should consider the opportunities working in partnership with the Student Union to offer volunteering and outdoor activities in a social prescribing style scheme. It is recommended that these spaces include physical features that facilitate socialising and studying as a priority. At the University of Sheffield specifically, there is a need to provide shelter from the noise and sight of the city, and accommodate for the poor weather during term time.

There is a need for green spaces which accommodate university students; spaces that are not seen as limited to members of the department associated with the nearest building. This facilitation should also be achieved through the spatial design. Students are focused on their university studies and socialising. It became apparent that lunch is the time university students take a break and are likely to seek an alternative environment. A successful green space would provide opportunities for both if it provided shelter from the weather and practical seating, which allows for both studying and social lunches.

### Limitations and Future Research

As accounted in the literature, there is a known influence to gender in the effect of connecting with nature in the workplace, if more data and resources had been available it would have been desirable to examine participants experiences through a more detailed lens. Future research should consider examining participants’ previous experiences with nature and the influence this has on their desire work and social spaces. There is opportunity to explore the role of gender and background on university students connection and engagement with urban nature.

It would also be interesting to further this area of research through a comparison of how these environments are measured in relation to green space, biodiversity and landscape architects design plans compared to how they are used across different universities. Future research should determine the value of trees in the immediate environment to university students to provide an accurate account of change in value when expanding campus buildings results in a loss of trees.

## Conclusion

This research focused on undergraduate university students (known as Generation Z), and as with other research into workplace design, the ability to implement behavioural change or create spaces which will be used by the target group relies on the ability of practitioners and decision makers to understand the realities of generational similarities and differences (Deal et al., 2010). To engage with university students in their requirements from the green spaces on campus requires consideration beyond the expected stereotypes. Therefore, translating this research’s findings into a real world application should be done with the collaboration of the intended user community.

The policy and practice implication from this research relates primarily to the use and design of nature-based interventions for university students within the university environment. Second, but no less crucial, are the broader implications for wellbeing interventions and urban green space development within the UK context.

Human health and wellbeing in the natural environment continue to develop as a field of research. Progress has been made in the spaces which are considered within this area, for example, the developed version of MENE (now known as People and Nature Survey) now includes questions on personal gardens. The King’s Fund policy brief on gardens and health highlights the importance of further integration of gardens into mainstream health practice (Buck, 2016). This approach should be taken in the evaluation of university campus design. As previously discussed, mental health issues reduce students’ attention and attainment; integrating spaces designed for students to use as study and social spaces could support a preventative approach to wellbeing on campus. The design of these spaces needs to consider the desired use of the space beyond the physical appearance, and as previously discussed campus space operates under social constraints similar to the workplace. Future research could trial the elements proposed in this research and investigate ways to create outdoor social and study spaces on campuses. Ultimately, it is about the integration of infrastructure and interventions into every day for students’ wellbeing.

## Data Availability Statement

The original contributions presented in the study are included in the article/Supplementary Material; further inquiries can be directed to the corresponding author.

## Ethics Statement

The studies involving human participants were reviewed and approved by the Department of Landscape in accordance with procedure laid down by the University of Sheffield’s Research Ethics Committee, which monitors the application and delivery of the University’s Ethics Review Procedure across the University, reference number: 016529 and 014504. The patients/participants provided their written informed consent to participate in this study.

## Author Contributions

The author confirms being the sole contributor of this work and has approved it for publication.

## Funding

This work was supported by the University of Sheffield as part of a research project funded by the Natural Environment Research Council, ESRC, BBSRC, AHRC, Defra, and IWUN [NE/N013565/1].

## Author Disclaimer

The views expressed in this publication are those of the authors and not necessarily of the funders or data providers.

## Conflict of Interest

The author declares that the research was conducted in the absence of any commercial or financial relationships that could be construed as a potential conflict of interest.

## Publisher’s Note

All claims expressed in this article are solely those of the authors and do not necessarily represent those of their affiliated organizations, or those of the publisher, the editors and the reviewers. Any product that may be evaluated in this article, or claim that may be made by its manufacturer, is not guaranteed or endorsed by the publisher.

## References

[ref1] Abdelaal M. S. (2019). Biophilic campus: An emerging planning approach for a sustainable innovation-conducive university. J. Clean. Prod. 215, 1445–1456. doi: 10.1016/j.jclepro.2019.01.185

[ref22] Active Fife (2019). Bums off seats. Available at: https://www.activefife.co.uk/bums-off-seats/ (Accessed January 18, 2020).

[ref2] Astell-Burt T. Feng X. (2019). Association of Urban Green Space With Mental Health and General Health Among Adults in Australia. JAMA Netw. Open 2:e198209. doi: 10.1001/jamanetworkopen.2019.8209, PMID: 31348510 PMC6661720

[ref3] Barton H. Grant M. (2006). A health map for the local human habitat. J. R. Soc. Promot. Heal. 126, 252–253. doi: 10.1177/1466424006070466, PMID: 17152313

[ref4] BBC (2019). Sheffield trees saved from felling after council U-turn - BBC News, BBC News Website. Available at: https://www.bbc.co.uk/news/uk-england-south-yorkshire-48934794 (Accessed July 29, 2019).

[ref5] Beckers R. van der Voordt T. Dewulf G. (2016). Learning space preferences of higher education students. Build. Environ. 104, 243–252. doi: 10.1016/j.buildenv.2016.05.013, PMID: 32859197

[ref6] Bell S. Thompson C. W. Travlou P. (2003). Contested views of freedom and control: children, teenagers and urban fringe woodlands in Central Scotland. Urban For. Urban Green. 2, 87–100. doi: 10.1078/1618-8667-00026

[ref7] Bell S. L.. (2014). Green space, health and wellbeing: making space for individual agency. Health Place 30, 287–292. doi: 10.1016/j.healthplace.2014.10.005, PMID: 25453749

[ref8] Benson M. Jackson E. (2013). Place-making and place maintenance: performativity, place and belonging among the middle classes. Sociology 47, 793–809. doi: 10.1177/0038038512454350

[ref9] Birch J. Risbeth C. Payne S. (2020). Nature doesn’t judge you – how urban nature supports young people’s mental health and wellbeing in a UK city. Health Place [Epub ahead of print] 62:102296. doi: 10.1016/j.healthplace.2020.102296, PMID: 32479372

[ref10] Bird W. (2007). Natural thinking. Royal Soc. protec. birds, 1–116.

[ref11] Boyd F. White M. P. Bell S. L. Burt J. (2018). Who doesn’t visit natural environments for recreation and why: A population representative analysis of spatial, individual and temporal factors among adults in England. Landsc. Urban Plan. 175, 102–113. doi: 10.1016/j.landurbplan.2018.03.016

[ref002] Boyd F. (2020). Tailoring Engagement with Urban Nature for University of Sheffield Students’ Wellbeing. PhD thesis, University of Sheffield.

[ref003] Boyd F. (2022). “University students noticing nature: the unpleasant, the threatening and the unfamiliar,” in Unfamiliar Landscapes 1st Edn. eds. Smith T. A. Pitt H. Dunkley R. A. (Cham: Palgrave Macmillan).

[ref12] Buck D. (2016). Gardens and Health: Implications for Policy and Practice. The King’s Fund, London.

[ref13] Cage E. Jones E. Ryan G. Hughes G. Spanner L. (2021). Student mental health and transitions into, through and out of university: student and staff perspectives. J. Furth. High. Educ. 45, 1076–1089. doi: 10.1080/0309877X.2021.1875203

[ref14] Campisi T. Caselli B. Rossetti S. Torrisi V. (2022). The evolution of sustainable mobility and urban space planning: exploring the factors contributing to the regeneration of car parking in living spaces. Transport. Res. Procedia 60, 76–83. doi: 10.1016/j.trpro.2021.12.011

[ref15] Charmaz K. (2006). Constructing Grounded Theory: a Practical guide through Qualitative Analysis. Thousand Oaks: Sage Publications.

[ref16] Colley K. Craig T. (2019). Natural places: perceptions of wildness and attachment to local greenspace. J. Environ. Psychol. 61, 71–78. doi: 10.1016/j.jenvp.2018.12.007

[ref17] Dallimer M. Davies Z. G. Irvine K. N. Maltby L. Warren P. H. Gaston K. J.. (2014). What personal and environmental factors determine frequency of urban greenspace use? Int. J. Environ. Res. Public Health 11, 7977–7992. doi: 10.3390/ijerph110807977, PMID: 25105548 PMC4143844

[ref18] de Vries S. van Dillen S. M. E. E. Groenewegen P. P. Spreeuwenberg P. (2013). Streetscape greenery and health: stress, social cohesion and physical activity as mediators. Soc. Sci. Med. 94, 26–33. doi: 10.1016/j.socscimed.2013.06.030, PMID: 23931942

[ref19] Deal J. J. Altman D. G. Rogelberg S. G. (2010). Millennials at work: what we know and what we need to do (If anything). J. Bus. Psychol. 25, 191–199. doi: 10.1007/s10869-010-9177-2

[ref20] Defra J. (2018). A Green Future: Our 25 Year Plan to Improve the Environment Exford, England Elsevier.

[ref21] Dunn R. R. Gavin M. C. Sanchez M. C. Solomon J. N. (2006). The pigeon paradox: dependence of global conservation on urban nature. Conserv. Biol. 20, 1814–1816. doi: 10.1111/j.1523-1739.2006.00533.x, PMID: 17181818

[ref23] Friends of Crookesmoor Parks (2020) Crookes Valley Park. Available at: https://crookesmoorparks.wordpress.com/about-the-parks/crookes-valley-park/ (Accessed January 28, 2020).

[ref24] Frumkin H. Frank L. Jackson R. J. (2004). Urban Sprawl and Public Health: Designing, Planning, and Building for Healthy Communities. Washington, D.C.: Island Press.

[ref25] Halpenny E. A. (2010). Pro-environmental behaviours and park visitors: The effect of place attachment. J. Environ. Psychol. 30, 409–421. doi: 10.1016/j.jenvp.2010.04.006

[ref26] Hartig T. (2006). Where best to take a booster break? American J. Prevent. Med. Elsevier. 31:350. doi: 10.1016/j.amepre.2006.06.003, PMID: 16979461

[ref27] Hartig T. Mitchell R. de Vries S. Frumkin H. (2014). Nature and health. Annu. Rev. Public Health 35, 207–228. doi: 10.1146/annurev-publhealth-032013-182443, PMID: 24387090

[ref28] Hipp J. A.. (2016). The relationship Between perceived greenness and perceived Restorativeness of university campuses and student-reported quality of life. Environ. Behav. 48, 1292–1308. doi: 10.1177/0013916515598200

[ref29] Historic England Archive (2004). Weston Park Sheffield. Available at: https://historicengland.org.uk/listing/the-list/list-entry/1001340 (Accessed January 28, 2020).

[ref30] Hitchings R. (2013). Studying the preoccupations that prevent people from going into green space. Landsc. Urban Plan. 118, 98–102. doi: 10.1016/j.landurbplan.2012.09.006

[ref001] Holt E. Lombard Q. Best N. Smiley-Smith S. Quinn J. (2019). Active and passive use of green space, health, and well-being amongst University students. Int. J. Environ. Res. Public Health 16:424. doi: 10.3390/ijerph16030424 PMC6388138 30717193

[ref31] Hughes G. Spanner L. (2019). The University Mental Health Charter. Leeds: Student Minds.

[ref32] Hughes J. Rogerson M. Barton J. Bragg R. (2019). Age and connection to nature: when is engagement critical? Front. Ecol. Environ. 17, 265–269. doi: 10.1002/fee.2035, PMID: 22271841

[ref33] Hunter M. C. R. Gillespie B. W. Chen S. Y. P. (2019). Urban nature experiences reduce stress in the context of daily life based on salivary biomarkers. Front. Psychol. 10:722. doi: 10.3389/fpsyg.2019.00722 31019479 PMC6458297

[ref34] Hunter R. F. Cleland C. Cleary A. Droomers M. Wheeler B. W. Sinnett D.. (2019). Environmental, health, wellbeing, social and equity effects of urban green space interventions: A meta-narrative evidence synthesis. Environ. Inter. Pergamon 130:104923. doi: 10.1016/j.envint.2019.104923, PMID: 31228780

[ref35] Ibrahim N. Fadzil N. H. (2013). Informal setting for learning on campus: usage and preference. Procedia Soc. Behav. Sci. 105, 344–351. doi: 10.1016/j.sbspro.2013.11.036

[ref36] Jones D. R. (2013). ‘The Biophilic university’: a de-familiarizing organizational metaphor for ecological sustainability? J. Clean. Prod. 48, 148–165. doi: 10.1016/j.jclepro.2013.02.019

[ref37] Kaplan R. (1993). The role of nature in the context of the workplace. Landsc. Urban Plan. 26, 193–201. doi: 10.1016/0169-2046(93)90016-7

[ref38] Lau S. S. Y. Gou Z. Liu Y. (2014). Healthy campus by open space design: approaches and guidelines. Front. Architec. Res. 3, 452–467. doi: 10.1016/j.foar.2014.06.006

[ref39] Liprini R. M. (2014). Students’ Perceptions of green space on a University campus: an Attention Restoration Theory Perspective. South Africa: University of Pretoria.

[ref40] Louv R. (2008). Last Child in the Woods. Second. Chapel Hill, North Carolina: Algonquin books.

[ref41] Lovell R. Depledge M. Maxwell S. (2018). “Health and the natural environment: A review of evidence, policy, practice and opportunities for the future”. 1–161.

[ref42] Lumber R. Richardson M. Sheffield D. (2017). Beyond knowing nature: contact, emotion, compassion, meaning, and beauty are pathways to nature connection. PLoS One 12:e0177186. doi: 10.1371/journal.pone.0177186 28486515 PMC5423657

[ref43] Maller C. Townsend M. Leger L. S. Henderson-wilson C. Pryor A. Prosser L.. (2009). Healthy parks, healthy people: The health benefits of contact with nature in a park context. The George Wright Forum 26, 51–83.

[ref44] Matloob F. A. Sulaiman A. B. Ali T. H. Shamsuddin S. Mardyya W. N. (2014). Sustaining campuses through physical character–The role of landscape. Procedia Soc. Behav. Sci. 140, 282–290. doi: 10.1016/j.sbspro.2014.04.421

[ref45] McDonald R. I. Beatley T. Elmqvist T. (2018). The green soul of the concrete jungle: the urban century, the urban psychological penalty, and the role of nature. Sustain. Earth 1, 1–13. doi: 10.1186/s42055-018-0002-5

[ref46] McEwan K.. (2020). Shmapped: development of an app to record and promote the well-being benefits of noticing urban nature. Transl. Behav. Med. 10, 723–733. doi: 10.1093/tbm/ibz027, PMID: 30834438

[ref47] McFarland A. L. Waliczek T. M. Zajicek J. M. (2008). The relationship Between student use of campus green spaces and perceptions of quality of life. Am. Soc. Horticult. Sci. 18, 232–238. doi: 10.21273/HORTTECH.18.2.232

[ref48] Milligan C. Bingley A. (2007). Restorative places or scary spaces? The impact of woodland on the mental well-being of young adults. Health Place 13, 799–811. doi: 10.1016/j.healthplace.2007.01.005, PMID: 17383927

[ref49] Moss S. (2012). “Natural Childhood,” London: National Trust.

[ref50] Natural England (2019). Monitor of Engagement with the Natural Environment: Headline Report 2019.

[ref51] Panno A. Carrus G. Lafortezza R. Mariani L. Sanesi G. (2017). Nature-based solutions to promote human resilience and wellbeing in cities during increasingly hot summers. Environ. Res. 159, 249–256. doi: 10.1016/j.envres.2017.08.016, PMID: 28822309

[ref52] Priestley M. Broglia E. Hughes G. Spanner L. (2022). Student perspectives on improving mental health support services at university. Couns. Psychother. Res. 22. doi: 10.1002/capr.12391, PMID: 35227409

[ref53] Pritchard A. Richardson M. Sheffield D. McEwan K. (2019). The relationship Between nature connectedness and Eudaimonic well-being: A meta-analysis. J. Happiness Stud. 21, 1145–1167. doi: 10.1007/s10902-019-00118-6

[ref54] Raymond C. M. Brown G. Weber D. (2010). The measurement of place attachment: personal, community, and environmental connections. J. Environ. Psychol. 30, 422–434. doi: 10.1016/j.jenvp.2010.08.002

[ref55] Redondo R. Valor C. Carrero I. (2021). Unraveling the relationship between well-being, sustainable consumption and nature relatedness: a study of university students. Appl. Res. Qual. Life 2021, 1–18. doi: 10.1007/S11482-021-09931-9

[ref56] Richardson M. Sheffield D. (2017). Three good things in nature: noticing nearby nature brings sustained increases in connection with nature / Tres cosas buenas de la naturaleza: prestar atención a la naturaleza cercana produce incrementos prolongados en conexión con la naturaleza. Psyecology 8, 1–32. doi: 10.1080/21711976.2016.1267136

[ref57] Richardson M.. (2019). A measure of nature connectedness for children and adults: validation, performance, and insights. Sustainability (Switzerland) 11:3250. doi: 10.3390/su11123250

[ref58] Sbaraini A. Carter S. M. Evans R. Blinkhorn A. (2011). How to do a grounded theory study: A worked example of a study of dental practices. BMC Med. Res. Methodol. 11:128. doi: 10.1186/1471-2288-11-128 21902844 PMC3184112

[ref59] Scannell L. Gifford R. (2010). Defining place attachment: A tripartite organizing framework. J. Environ. Psychol. 30, 1–10. doi: 10.1016/j.jenvp.2009.09.006

[ref60] Scannell L. Gifford R. (2017). The experienced psychological benefits of place attachment. J. Environ. Psychol. 51, 256–269. doi: 10.1016/j.jenvp.2017.04.001

[ref61] Seaman P. J. Jones R. Ellaway A. (2010). It’s not just about the park, it’s about integration too: why people choose to use or not use urban greenspaces. Int. J. Behav. Nutr. Phys. Act. 7:78. doi: 10.1186/1479-5868-7-78, PMID: 21029448 PMC2978120

[ref62] Shanahan D. F. Bush R. Gaston K. J. Lin B. B. Dean J. Barber E.. (2016). Health benefits from nature experiences depend on dose. Sci. Rep. 6:28551. doi: 10.1038/srep28551 27334040 PMC4917833

[ref63] Southon G. E. Jorgensen A. Dunnett N. Hoyle H. Evans K. L. (2018). Perceived species-richness in urban green spaces: cues, accuracy and well-being impacts. Landsc. Urban Plan. 172, 1–10. doi: 10.1016/j.landurbplan.2017.12.002

[ref64] Speake J. Edmondson S. Nawaz H. (2013). Everyday encounters with nature: students’ perceptions and use of university campus green spaces. Hum. Geogr. 7, 21–31. doi: 10.5719/hgeo.2013.71.21

[ref65] Tyrväinen L. Ojala A. Korpela K. Lanki T. Tsunetsugu Y. Kagawa T.. (2014). The influence of urban green environments on stress relief measures: A field experiment. J. Environ. Psychol. 38, 1–9. doi: 10.1016/j.jenvp.2013.12.005

[ref66] White M. P. Wheeler B. W. Herbert S. Alcock I. Depledge M. H. (2014). Coastal proximity and physical activity: is the coast an under-appreciated public health resource? Prev. Med. 69, 135–140. doi: 10.1016/j.ypmed.2014.09.016, PMID: 25284259

[ref67] Windhorst E. Williams A. (2015). ‘It’s like a different world’: natural places, post-secondary students, and mental health. Health Place 34, 241–250. doi: 10.1016/j.healthplace.2015.06.002 26093082

[ref68] Zhang J. W. Howell R. T. Iyer R. (2014). Engagement with natural beauty moderates the positive relation between connectedness with nature and psychological well-being. J. Environ. Psychol. 38, 55–63. doi: 10.1016/j.jenvp.2013.12.013

