# Supplementary material for: Between the Library and Lectures: How Can Nature Be Integrated Into University Infrastructure to Improve Students’ Mental Health
Source: Front Psychol. 2022 Jun 17;13:865422. doi: 10.3389/fpsyg.2022.865422 (PMC9252603; doi:10.3389/fpsyg.2022.865422)
Supplement: Supplementary file 1 [file Data_Sheet_1.DOCX]

Supplementary material

**Supported nature connection theories**

Biophilia hypothesis: This is the innate tendency to an affiliation with the natural environment. Introduced by Wilson in 1984, this hypothesis is founded on nature and humans being unequivocally connected (Wilson, 1984). In recent years, the idea of biophilia in design and architecture has gained popularity, whereby the built environment is designed in synthesis with nature through the integration of plants, landscape design and use of natural form (McDonald, Beatley and Elmqvist, 2018).

Attention Restoration Theory: Prolonged levels of mental engagement result in directed attention fatigue (Kaplan, 1995). According to Kaplan (1995) nature provides an environment that allows for recuperation because it allows the mind to ‘get away’ from the usual habits by providing ‘soft fascination’ through natural phenomena, such as clouds moving. Being in the natural environment can facilitate a neutral space in which a person may experience respite, unlike in built spaces which are more likely to contain predefined standards and societal expectations (Kaplan, 1995). The benefit of attention restoration can also be experienced in micro-form or through the addition of natural elements to indoor settings. The use of indoor plants or views of nature from a window provides opportunity for the mind to recuperate. Application of ART to indoor spaces have seen a positive effect on stress and fatigue (Kaplan, 1993).

Psycho-evolutionary Stress Reduction Theory: Natural environments offer specific attributes inherent to survival that humans have evolved to have a preference for, such as water and open spaces (Ulrich *et al.*, 1991). Originated in Ulrich’s research on hospital recovery, it has been found that exposure to the natural environment produces a salient parasympathetic nervous system response which promotes a positive emotional state and physiological activity, which create a sustained attention and perceptual intake (Ulrich, 1984; Ulrich *et al.*, 1991).

This research is founded in the role of Attention Restoration Theory (ART) in nature improving respite from university life. ART and Psycho-evolutionary Stress Reduction Theory can be considered parallel theories that explain the related human cognitive and affective response to nature (Ulrich, 1984; Kaplan, 1995; Berto, 2014). The biophilia hypothesis offers an overarching principle to humans’ relationship with the natural environment (Wilson, 1984).

Descriptions of Metrics

Recovering Quality of Life (ReQoL)
Recovering Quality of Life (ReQoL-10) is 10-item self-reported recovery focused quality of life measure (Keetharuth *et al.*, 2018). It contains 10 questions on mental health and one on physical health. This self-reported outcome measure is designed to comprehend the quality of life of someone with a mental health condition. It is a development on the Short Warwick-Edinburgh Mental Wellbeing Scale and EQ-5D with a simple and accessible question format. It is designed to be consistent with themes of recovery (hope, activity, belonging, relationships etc.) and is suitable for a range of mental health conditions including common mental health disorders such as depression and anxiety.

Nature Relatedness (NR-6)
Shmapped included the short form version of the Nature Relatedness scale that assess the affective, cognitive, and experiential aspects of individual’s connection to nature. It has been validated with respect to an assortment of environmental and personality measures (Nisbet, Zelenski and Murphy, 2009). Nature relatedness is a useful measure for understanding a person’s relationship with nature and the processes underlying environmental concern and behaviours (Nisbet, Zelenski and Murphy, 2009).

Inclusion of Nature in Self (INS)
The Inclusion of Nature in Self scale is based on the theoretical foundation that the characteristics of the natural environment can be used for self-benefit and therefore self-nature connection can be defined as ‘the extent to which an individual includes nature within [their] cognitive representation of self ‘ (Wesley Schultz, 2001, Schultz, 2002 p.67). It is concise and composed of seven images that depict a venn diagram of ‘self’ and ‘nature’; these circle, become closer together to the point of being one.
